# How do family members influence smokeless tobacco consumption during pregnancy in India? Perspectives of pregnant women

**DOI:** 10.1371/journal.pgph.0002828

**Published:** 2024-06-20

**Authors:** Radha Sharma, Jaishree Ganjiwale, Mona Kanaan, Kate Flemming, Kamran Siddiqi

**Affiliations:** 1 Department of Health Sciences, University of York, York, United Kingdom; 2 ConnectHEOR Canada Limited, Edmonton, Canada; 3 Central Research Services & Department of Community Medicine, Pramukhswami Medical College, Karamsad, India; 4 Hull York Medical School, University of York, York, United Kingdom; London School of Hygiene and Tropical Medicine Faculty of Epidemiology and Population Health, UNITED KINGDOM

## Abstract

Smokeless tobacco (ST) use in South Asia is culturally ingrained and socially accepted. A better understanding of these sociocultural influences could inform behavioural approaches to prevent ST use. We sought to understand how family members influence pregnant women’s behaviour, attitudes, and perceptions towards ST use. Moreover, we captured the influence of community health workers in this context. A qualitative study using a framework analysis was conducted in selected Indian populations. Eight in-depth interviews among pregnant and postpartum women were conducted in Gujarati, the local language, investigating ST use during pregnancy and the influence of family and peers. All transcripts were transcribed verbatim and translated into English and analyzed in NVivo. The social norms and expectations around ST during pregnancy appeared to have shifted away from promoting towards discouraging its use in the past few years. Women described how their spouses and other family members encouraged them to stop using ST during pregnancy, with some women must hide their ST use from their family members. They also received advice on the harms of ST use from community health workers (Accredited Social Health Activist–ASHA workers). Influenced by the advice received from such workers, several women tried to reduce their ST use during pregnancy. Our findings suggest that the acceptability of ST use in pregnancy may be in decline among families in India. Hence, efforts to promote ST prevention during pregnancy are likely to be “pushing against an open door”. Furthermore, community health workers appeared to play an influential role in supporting women to abstain from ST use during pregnancy.

## Introduction

Smokeless tobacco (ST) is used by more than 356 million adults worldwide; most of these consumers live in low and middle-income countries (LMICs), especially South Asia [1]. Despite the widespread use of ST in nearly 140 countries, research in this area is limited, especially focusing on women and pregnancy.

ST use is associated with higher incidences of head and neck cancers, cardiovascular mortality, and possibly poor birth outcomes [1–4]. Its prevention and control is challenging due to the diversity of ST products, its complex supply chain and related behaviours, its social acceptance and cultural integration [5–7]. In the South-East Asia Region (SEAR), ST is a cheaper and socially acceptable alternative to smoking, which makes it popular among females [8–10]. At times, it has been used to treat common pregnancy-related ailments such as morning sickness and a bitter taste in the mouth [11, 12]. A better understanding of the sociocultural influences of ST use during pregnancy is required to inform culturally appropriate interventions to prevent and control ST use.

There is some evidence that family influences ST use during pregnancy [9, 11–13]. It is reported that women are more likely to use ST if their relatives, especially their husband, use ST. This is supported by the review conducted by Kakde and colleagues (2012) which highlighted that family and friends tend to act as both facilitators and barriers to ST use. To further strengthen the evidence of family influence, Begum and colleagues (2015) reported several narratives by women which referred to family members being influential in the initiation of their ST habit [13].

The prevalence of current smokeless tobacco use among adults as per the Global Adult Tobacco Survey (2016/17) in India is estimated at 24% (13% among women and 30% among men), with about 11% of women using smokeless tobacco daily [14]. Several smokeless tobacco products are used in India, including betel quid with tobacco, khaini, gutka, oral tobacco, paan masala, and snuff. Among women residing in rural areas, the use of khaini (tobacco lime mixture) and gutka (tobacco, line, areca nut mixture) is most common, 13.5% and 7.1%, respectively [14].

The cultural acceptance of ST use in SEAR and its prevalence among elderly family members suggest that family plays a significant role in shaping women’s ST use behaviour [11]. Therefore, preventive/cessation measures related to ST use during pregnancy need socio-cultural adaptation, including influences from family members [8]. Hence, exploring the “how” factor is important to understand family influence on ST use during pregnancy, and to further, if possible, modify these influences for tobacco control measures. The aim of this study is to understand how family members influence pregnant women’s behaviour, attitudes, and perceptions towards ST use and further gather insights into the perspectives of pregnant women themselves towards ST use during pregnancy.

## Methodology

### Setting and design

This qualitative study was conducted in Bhadran village, in the province of Gujarat, India. It is a rural area with a primary health centre (PHC) that delivers antenatal and basic health services via the community health workers (CHW), “Accredited Social Health Activist” (ASHA–trained female community health workers). The prevalence of ST use among adults in the province of Gujarat is 19.2% [14]. This is comparable to the national prevalence of 21.4% (Global Adult Tobacco Surveys (GATS) India, 2018) [14]. Personal communication with the medical officer at the PHC indicated that the prevalence at the village level is similar to that of the province, at the time of the study.

### Recruitment and sampling

Using purposive sampling, we recruited pregnant or postpartum women (up to 6 weeks after birth), who consumed ST at any point in their pregnancy. The identification of eligible women was carried out by CHWs, but they did not directly approach them to participate in the study. The researcher (RS) approached the identified eligible women and obtained informed consent for their voluntary participation in the study. Women were given the option to choose the interview location where they felt most comfortable.

### Honorarium

Participants were given a small incentive (100 Indian rupees (~1.3 USD)) to appreciate their time for the interview in the form of a prepaid calling card for local talk time.

### Data collection

In-depth interviews were conducted in Gujarati, the local language, between November and December 2019 using a topic guide (S1 Table). The development of the topic guide was largely informed by an existing systematic review, [15] and with input from the healthcare providers at the selected PHC in India. This included capturing the direct (encouraging or discouraging) and indirect (use within the family) influence of family members on ST use, its use within pregnancy (reasons for use, perception, initiation), and the knowledge and beliefs related to ST use during pregnancy. Prior to its use in the study, the topic guide was piloted with three participants and modified accordingly. Interviews were conducted by one of the researchers (RS). The interviews were audio recorded and imported to secure university storage as soon as possible.

### Data analysis

**Fig 1:**
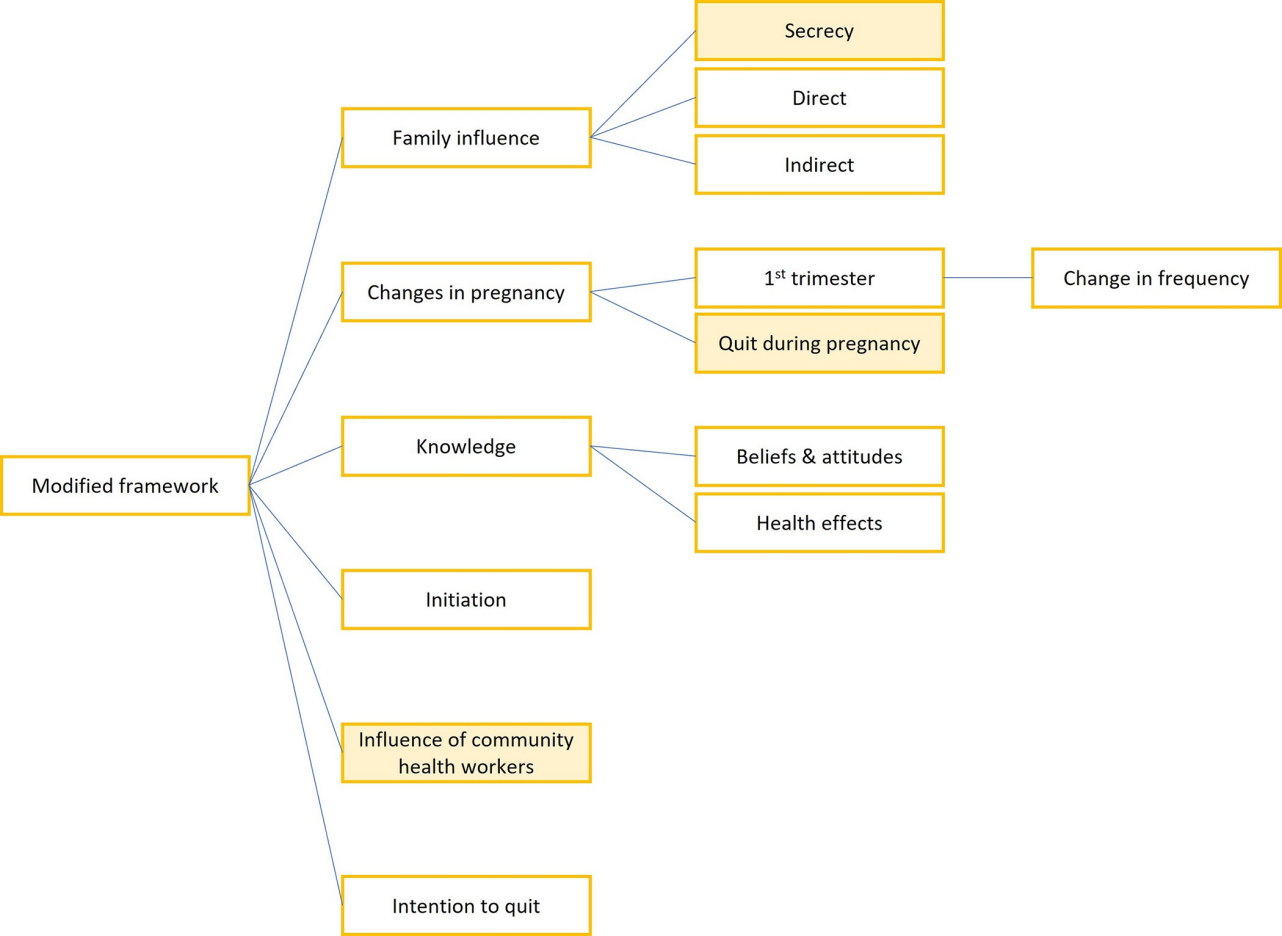
Framework adapted for this qualitative study based on the interview transcripts.

This study was analysed using the framework analysis method [16]. The interviews were translated and back-translated to ensure that the context in the local language was retained. A preceding mixed-method systematic review exploring the socio-cultural context of ST use among women primarily aided the framework development [15]. Additionally, the inductive open coding method, allowed modification of the framework (*Fig 1*) for this study as new themes emerged from the in-depth interviews that didn’t fit the framework. An inductive approach is an unrestricted form of open coding where new/emerging concepts from the data are captured and later refined into themes [16, 17]. Changes from the preceding framework were the addition of two new themes (influence of CHWs and intention to quit, and a new sub-theme of secrecy under the theme of familial influence).

NVivo data management software, [18] was used for coding, development of framework matrix and data management.

### Ethics

The study received ethical approval from the University of York Research Governance Committee (UK) and the Foundations for Diffusion of Innovations Ethics Committee (FDIEC) (India). Formal written consent was obtained from each participant.

## Results

**Fig 2:**
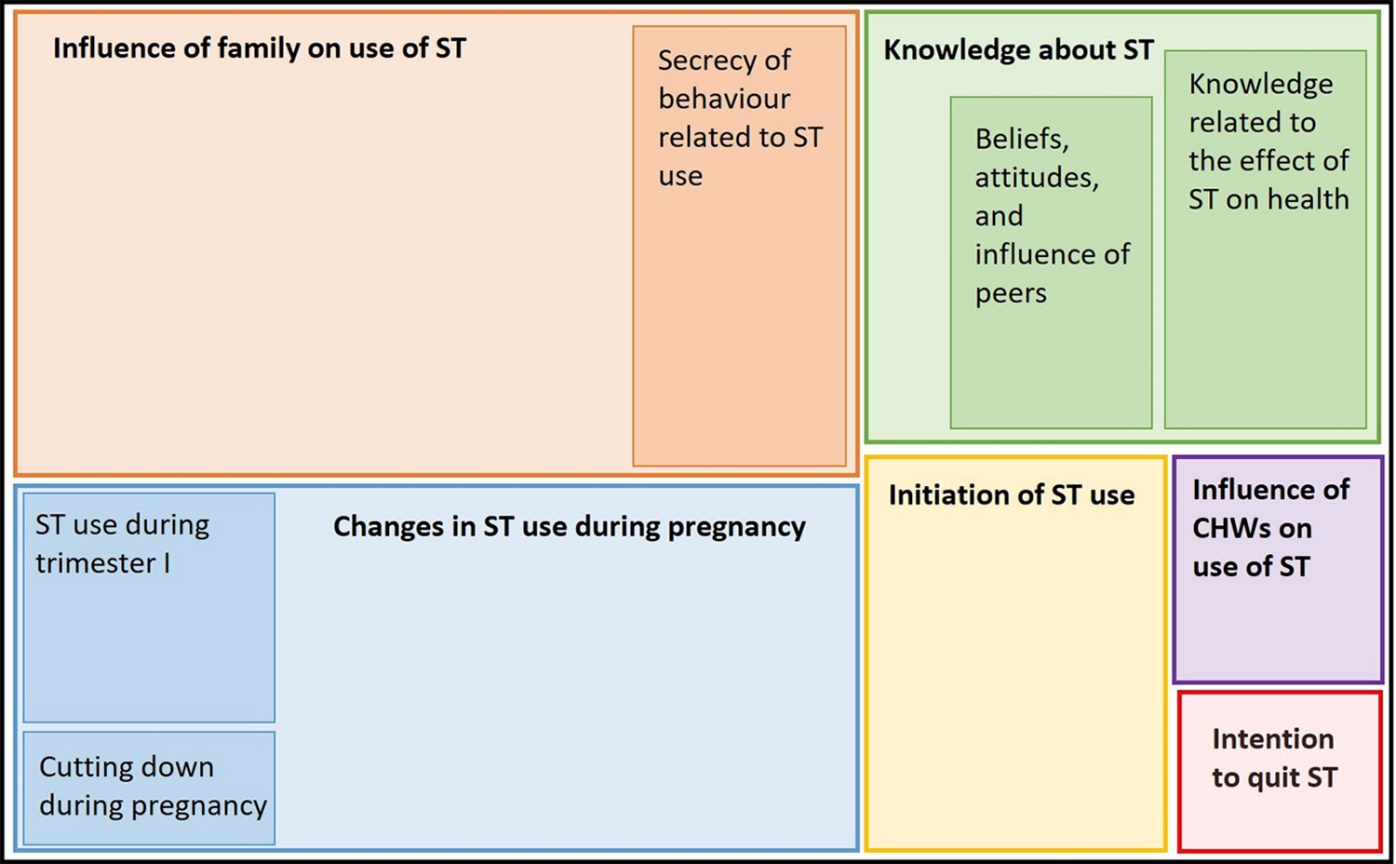
Hierarchy chart of the codes/themes from the framework analysis.

A total of eight in-depth interviews were conducted and findings from the analysis are summarized below (*Fig 2*) in a framework hierarchy chart. Six major themes were identified; 1. Initiation of ST use, 2. Knowledge about ST, 3. Influence of family on ST use, 4. Change in ST use during pregnancy, 5. Influence of CHWs on ST use, and 6. Intention to quit. These were further divided into sub-themes (*Fig 2*). The themes and sub-themes are detailed below.

### Theme 1: Initiation of ST use

Conversations related to the initiation of ST use among these women were around various aspects. In addition to the influence of family on initiation of ST use (theme 3), it was noted that the habit is formulated in early childhood for most women (n = 5), while for some women the first use of ST was during adolescent age (n = 3). Participants could not recall an exact age; however, its use for a very long time, ever since childhood or reference to high school graduation or other life events were mentioned for approximate age.

*“Ever since I was like this (referring to her firstborn, who is around 4 years), since then.” (26 years, 6 weeks post-partum)*

“Must be small… 5–6 years.” (33 years, 5 months pregnant)

While most of the participants initiated the use of ST in early childhood, one of the participants started using ST during the adolescent phase.

*“Long time, ever since I finished 12th grade… since then.” (25 years, 5 months pregnant)*

Additionally, women often mentioned about initiating the use early in childhood/teen years as an influence of peers while performing routine chores.

*“For no particular reason*… *like this that I go to the farm right*, *so everyone eats*. *So that way started eating it (participants often mentioned “eating it”*, *which meant consuming ST*) *and then go addicted to it*.*” (25 years*, *5 months post-partum)*.

The habit perhaps also started by experimenting or trying as it was consumed by everyone in the community surroundings of these women.

*“Everyone was eating. So, 2–2 dana (dana is a local term which refers to small pinch like portions. To not take away from the meaning, the actual word is used as it is.) I ate. So, from that got habituated.” (23 years, 7 months pregnant)*

### Theme 2: Knowledge about ST

#### 2a: Beliefs, attitudes, and influence of peers

The general belief that consuming ST is normal and that it will cause no harm was mentioned by participants (n = 5). This was often mentioned in the context of peers influencing the use of ST, and persuading that it is completely fine to do so.

*“So, all our friends that are there right. They all say, nothing will happen, just eat it…. So, I would put it in my mouth and it would feel nice. So then again, I would get it myself and eat it, like that.” (25 years– 5 months pregnant)*

The use of ST as an activity to kill boredom was also mentioned by one of the participants. Furthermore, the extent of ST use within the community highlights how normal and acceptable it is.

*“This when I am not able to pass time so then I eat it. So, let me tell you. If anyone wants to eat they can eat. Who doesn’t eat right now, everyone has it in their mouth.” (26 years– 8 months pregnant)*

It was observed during the majority of the interviews that the use of ST was initiated because of peer and/or community influence. That is, it seemed so normal and common to women when they started the use of ST and that was related to the belief that there is no harm associated with it.

*“This everyone before use to eat right, so little portions I also put in my mouth. From that got addicted.” (25 years– 8 months pregnant).*

*“To me, it is like, everyone is eating it then I also put small portions in my mouth… that’s what I feel.” (23 years– 7 months pregnant)*

#### 2b: Knowledge related to the effect of ST on health

Participants generally were aware that the ST pouches (referred to as *Vimal [gutka—tobacco*, *lime*, *and areca nut mixture]*, which is a brand name, and that is how it is locally identified) contain tobacco and other additives. However, most were not aware that tobacco is a harmful substance in general. This lack of awareness was mainly when they had started the use of ST at an early age.

*“If I had known, why would I have eaten it.” (26 years, 8 months pregnant)*

*“Everyone say that this is “Vimal” like that … even if you ask a little child, they know about this. Inside, it has areca nut, and tobacco and then mix it all. That much I know.” (24 years, 8 months pregnant)*

Over a period of time there was an increase in awareness and realisation that what is consumed in form of these ST pouches is actually harmful. This increase in awareness was either via family influence (in one of the narratives reported below) on general health related harmful effects of ST or through the CHWs educating about potential complications or outcomes related to ST use during pregnancy (quote mentioned in theme 4c “cutting down during pregnancy”). The awareness of- the health impacts of ST during pregnancy appeared to be a key influential factor as it led to some quit attempts among women (discussed further in theme 4c, “cutting down during pregnancy”).

*“He (husband) says forget about it … “Vimal” is not nice, it causes harm.” (26 years, 6 weeks post-partum)*

### Theme 3: Influence of family on use of ST

The influence of family members was either encouraging or discouraging the woman’s use of ST.

The use within the household and among close family members, influenced the use of ST among women from a very young age. It was noted that parents who were using ST, offered it to their children as young as 3 years of age.

A participant elaborated on how her father’s habit of using ST got her initiated towards the same whilst within the same family her mother was opposing the use of it. She further added that her husband also discourages her use of ST now, however due to the addictive nature of the habit she is unable to stop.

*“So, my father use to eat right, so I also ate a little and then I learnt using it. My mother use to yell at me for it, but my father would sneakily give it to me……. My husband knows, and he tells me a lot to forget about it but I am not able to stop using it” (26 years, 6 weeks post-partum)*

Similarly, other participants also mentioned about their spouses and other family members encouraging them to stop using ST.

*(talking about her husband suggesting stopping ST use) “It gives me a little bit of tension. So, then it feels like to stop using it.” (25 years, 5 months pregnant)*

*“My mother-in-law says, and my husband also says to stop, but I am not able to quit. If I want to stop, I really feel like stopping. Many times, I have thought that I won’t eat, but then still I feel like it and get tempted to eat it because until I don’t eat it, I feel very uneasy. Don’t feel like doing any work, nothing feels nice, and then the moment I put a little portion in the mouth, quickly I get all my work done.” (25 years, 8 months pregnant)*

However, the participant further adds that despite her husband suggesting stopping the use of ST, his opinion doesn’t matter, as the spouse himself consumes ST.

*“He doesn’t affect me. He eats it himself when he goes to the town … in the house he never asks or tells me if I want to consume it.” (25 years, 8 months pregnant)*

On the other hand, there was evidence that a strong family influence in the form of family pressure led to a woman quitting ST during her first pregnancy but eventually, during her second pregnancy, continued using ST without being noticed by her family.

*Before, then I use to not consume it. My father had made me swear by God (a personal belief)… .… So, the first one, my elder son time, I use to not use it. This time I am, but not a lot.” (24 years, 8 months pregnant)*

The influence of family members through this study appears to be mostly nudging, either in terms of using ST or suggesting stopping its use. A strong persuasive influence also comes across, especially with quitting the use of ST during pregnancy.

#### Theme 3a: Secrecy of behaviour related to ST use

One of the new themes that emerged was the secret behaviour related to ST use. The participants mentioned that no one in the family was aware of their ST habit and that it was something they consume secretly while doing their routine housework. Some exceptions were where a few participants mentioned that only their spouses were aware of the ST use within the family.

*“No, no one in the house knows. No even my father. No one” (23 years, 7 months pregnant)*

*“If my father finds out, he will yell at me. This I eat sneakily while doing work.” (25 years, 5 months pregnant)*

*“Not even my father-in-law… my mother-in-law consumes that also my father-in-law does not know.” (26 years, 8 months pregnant)*

However, this secret behaviour seems to be within the family only, as other women/peers who often work together in the community were aware of each other’s ST use.

#### Theme 3b: Accessibility of ST

The influence of family in relation to the accessibility of ST products was also noted. Women mentioned that they were often supplied the ST packets by family members. This contrasts with the previous theme of secret behaviour of ST use. One of the participants mentioned that she only receives the ST packets from her husband and if he doesn’t get it for her, she won’t go out to buy it and content herself with other food items.

*“If my husband provides only then … what to do, I am habituated now so. Can’t do without it.” (25 years, 5 months pregnant)*

Similarly, another participant mentioned that she craves ST to such an extent, that even an hour without it makes her irritable and, in a mood, to fight; as a result, her father-in-law will get her the ST packet.

*“If I don’t eat it for even half hour, then I feel like fighting with someone. I am just not able to stop it. I end up fighting with my father-in-law, then he brings it for me … an hour I go without using it, I become all flighty.” (26 years, 6 weeks post-partum)*

### Theme 4: Changes in ST use during pregnancy

The women who were interviewed were at different time points during their pregnancy, including those who had just delivered. Hence, the responses captured their overall change in frequency of ST use and attempt to quit during pregnancy. Additionally, participants were asked if there were any changes in their ST use in all three trimesters and the only changes, they reported were in the first trimester.

#### 4a: Change in frequency

Some women either decreased (n = 3) or increased (n = 2) their use of ST at some point during their pregnancy while for some ST use during pregnancy was similar to pre-pregnant state (n = 3).

A participant reported that her use of ST increased compared to the initial months of the pregnancy due to the watery sensation in the mouth (saliva build-up in the mouth, which is often a symptom experienced by pregnant women).

*“During the initial 2–3 months of the pregnancy, I use to eat it less … and then later as months progressed right. So, then I started consuming a little more … that watery feeling in the mouth happens right. So, I feel like to eat something. So, then I eat little portions of the this and so it has increased …. this watery feeling in the mouth, so that tempts to eat. What I can eat so that it feels good.” (25 years, 8 months pregnant)*

*“During the first pregnancy, I use to eat a lot of it. My daughter the first born right, that one year I use to eat a lot. One pouch I get, that will last for 2 days or 3 days. It’s not like I won’t eat it and it’s also not like I keep eating it … little portion I put and then again it feels like that in the mouth, so then I gargle it off. Then again pop in little portions in the mouth.” (26 years, 8 months pregnant)*

The decrease in the use of ST was primarily due to the influence of CHWs and this is further discussed in a later theme.

*“Before, I use to eat about 2-3-4 times a day. Now I have reduced a lot as madam said to not eat (referring to the CHW) … once in a while when I feel like it, I eat it.” (24 years, 8 months pregnant)*

Participants also mentioned that there was absolutely no change during pregnancy in their use of ST. This was noted not just during their current pregnancies, but also when reflecting back on their previous pregnancies.

*“During all three pregnancies I use to eat (ST). Not a lot, no increase, no decrease.” (26 years– 6 weeks post-partum)*

#### 4b: ST use during trimester I

Participants often reported that their use of ST during the initial months of the pregnancy was similar to, and a continuation of, their use from the pre-pregnancy phase. This was reported in multiple ways; either of them clearly stating that their use was similar or reporting similar quantities when specifically asked about the quantity consumed before and during the pregnancy. Additionally, it was also noted that the use of ST either increased or decreased in the first trimester in response to pregnancy-related symptoms.

*“With these pills (medicines taken as part of ante-natal care), my mouth feels bitter right. So, then I pop a little in my mouth.” (24 years, 8 months pregnant)*

On the flip side, the same participant reported that her use of ST was relatively less due to nausea during the first trimester and that ST triggered nausea.

*“No, there was no change as such. Initially during the pregnancy, that time “Vimal” doesn’t taste that good the tobacco one. Feels like vomiting. So, can’t eat a lot. As soon as I try to eat it, it would feel like vomiting so didn’t eat then … then some day when I feel like tit, I ate it.” (24 years, 8 months pregnant)*

Participants also often mentioned having decreased their use of ST mostly later in their pregnancy. These were related to potential harms ST could have on their pregnancy outcome and/or health in general and specifically related to trimester changes.

#### 4c: Cutting down during pregnancy

Across various interviews, it came across that women attempted to consciously either reduce their use of ST or quit during the pregnancy phase. This was mainly due to the influence and constant motivation of the CHWs. It seems that they tried to cut down on their use of ST due to the potential harms or complications it can cause during pregnancy.

*“Thoughtfully I have decreased my use. Because blood is less right in my body. So, I don’t eat it now. Then, at the time of delivery, they say of transfusion. So that’s why, I have myself decreased the use. And don’t eat it.” (23 years, 7 months pregnant)*

Additionally, a participant also reported that perhaps quitting now for the benefit of the child and eventually completely quitting for her own good. Hence, realising that ST not only would affect her child but is also harmful for her own health.

*“For the child … and now if I can stop it then it’s good, right …. I always feel like it but won’t eat it.” (25 years, 5 months pregnant)*

### Theme 5: Influence of CHWs on use of ST

A new theme that stood out in the majority of the interviews was the influence of healthcare workers on the use of tobacco among pregnant women. Participants often shared that they were trying to reduce the use of ST during their pregnancy based on the advice of the CHWs.

*“Till 6 months of pregnancy*, *I was eating this*. *Then*, *I stopped*. *The sister (referring to the* CHW*) use to tell me a lot so that’s why*.*” (22 years*, *4 weeks post-partum)*

*“Before, I use to eat about 2-3-4 time a day, now I have reduced a lot as this madam said to not eat (referring to the community health worker)… . once in a while when I feel like it, I eat it.” (24 years, 8 months pregnant)*

It was also noted that CHWs often shared extreme worst-case scenarios (some of which, were potentially not evidence-based) with pregnant women to encourage them to quit the use of ST during pregnancy. One of the post-partum participants shared the reason for which she quit the use of ST during her pregnancy.

*“Everyone use to say these ladies and all that if I don’t stop, they will have to transfuse blood and all. Maybe I have to have C-section. So, then I stopped.” (22 years, 4 weeks post-partum)*

### Theme 6: Intention to quit ST

In addition to an earlier theme about “quitting during pregnancy,” women also mentioned about the intention to quit in general. That is either attempting to quit before the pregnancy or desire to quit during the pregnancy and maintain it after the pregnancy as well. These previous or current quit attempts were without the support of any cessation aids such as quit lines.

*“Yes, that if I can slowly reduce the use … so that way can stop it … so won’t cause problem … and can stop it right.” (25 years, 5 months pregnancy)*

Furthermore, one of the participants had tried to quit the use of ST before marriage and started again afterwards.

*“I had tried before… But then I came here (post marriage) and started again.” (33 years, 5 months pregnancy)*

## Discussion

The findings of this study are important to understand how spouses, families, peers and CHWs influence the use of ST among pregnant women. We discuss the three key findings; attitudes and perceptions related to ST use during pregnancy, familial influence on its use, and the integration of CHWs in the prevention and cessation of ST use.

### Attitudes and perceptions related to ST use during pregnancy

It has been previously suggested that women initiate tobacco use often during pregnancy for various perceived benefits to combat pregnancy-related symptoms such as nausea, constipation, vomiting [11, 12]. However, none of the participants in the current study started ST use during their pregnancy. Though ST use increased for some participants mainly to combat common pregnancy-related symptoms, there was also an instance of a decrease in use due to nausea and vomiting during pregnancy. Furthermore, contrary to an earlier perception about ST use being acceptable in pregnancy, it would appear from the views expressed by the women in the study that this may be changing.

### Familial influence on ST use

The use among family members has influenced the use of ST among women from a very young age. This is consistent with the literature, that about 17% of the women in India (2018), start the use of ST before the age of 15 years and that the ST initiation is often linked to purchase activities for family members in early childhood [14, 19]. The current study also suggested that parents who were using ST, offered it to their children and that even during pregnancy women were supplied the ST packets by family members. In India, the odds of using ST among women are much higher if family and friends use ST (odds range from 2.1–5.0) [20]. It was noted that participants mentioned their spouse and other family members, suggesting stopping their use of ST during pregnancy. The influence of family on ST use among women, such as offering ST products during childhood and also suggesting its stopping during pregnancy or in adulthood, could potentially be due to the changing perception and increase in awareness about the harms of ST use. Tobacco control programs in India aim to increase awareness related to harmful effects of tobacco which could have contributed to this shift in perception [21]. This opens a new dimension to tobacco control among women in LMICs, where family members are discouraging the use of ST during pregnancy and hence a possible opportunity to augment this support through interventions. However, the study also highlights that even though the partner suggests stopping ST use during pregnancy, it potentially is not a strong influence if the partner uses ST himself. This is an important finding as it mirrors the views of women who continue to smoke during pregnancy when the partners ask a woman to stop smoking whilst continuing the habit themselves [22]. Furthermore, with respect to tobacco smoking, it is reported that quitting while living with a partner who smokes as well makes it more difficult and perhaps their behaviour acts as a barrier [22]. This potentially translates to ST use as well, given that women feel when others use it, why cannot they? This highlights the need to address ST control measures on a community level and the inclusion of family in the intervention delivery. There is some evidence of multi-component interventions, including family support that have been effective for smoking cessation, highlighting that family-based interventions along with another method can be combined together to increase intervention efficacy [23, 24]. Furthermore, a study specifically highlighted the preferences among smokers with severe mental illness on ways family can support their cessation efforts [25]. These components can be utilized to develop family-based interventions for smokeless tobacco use among women.

### Integration of CHWs in tobacco control measures

This study highlights the potential role of CHWs in tobacco control interventions, especially during pregnancy as ASHA workers were successful in supporting pregnant women to quit or reduce their ST use. Although the delivery of information on the harms of ST use during pregnancy could be improved, CHWs were effective in helping women quit or reduce their ST use. To enhance the effectiveness of counselling, it is important to identify the needs and support required by CHWs to deliver tobacco control interventions consistently and efficiently. One possible approach to consider is the use of the 5A screening tool (five-step screening tool designed for health care providers to identify and offer smoking cessation support), which could be adapted, and assessed for feasibility, and acceptability in integrating CHWs in offering cessation interventions for pregnant women who wish to quit or reduce their ST use during pregnancy [26]. Some studies have previously explored the integration of CHWs in tobacco control interventions [27–29]. One such study found that about 1/3 of CHWs informed patients about the harmful effects of tobacco to all patients, while about half provided information to patients suffering from specific illnesses. Furthermore, CHWs with training in tobacco control were more likely to provide information on adverse reproductive outcomes (OR 2.1) [28]. However, the knowledge among CHWs for harms related to adverse reproductive outcomes was low (4–10%). This was consistent with another study examining the knowledge of CHWs which reported that most (64%) CHWs had low knowledge about tobacco products and its consequences but had a positive attitude (86%) towards tobacco control [29].

Under national guidelines, the training of CHWs includes spreading awareness on tobacco as a risk factor for non-communicable diseases (NCDs) and to some extent its cessation during pregnancy, but additional training can be provided for smokeless tobacco prevention and control among women to prevent adverse reproductive outcomes [30]. This is especially important given that only 2.7% of women who attempted to quit ST opted for pharmacotherapy and about 8% opted for counselling as per the national survey(2016–2017) [14]. The majority of women attempted to quit ST without assistance due to low support for cessation and hesitancy to access cessation centres or quit lines. If pregnancy can be a motivating factor and these women are offered appropriate support, quit attempts and success can potentially be enhanced. Therefore, the integration of CHWs into tobacco control programmes, and family involvement and support shows promise for promoting ST cessation during pregnancy.

*The study has several strengths*. Firstly, the diversity of the participants really added to the richness of the data. Especially inclusion of postpartum women allowed us to capture how ST use was throughout the pregnancy and whether the changes during pregnancy persisted or changed (if at all). This enriched the study by understanding that pregnancy was a motivation to reduce/quit ST use and once the pregnancy ended, so did the motivation and hence the participant started ST use as a pre-pregnant state. Secondly, the interviews were able to capture the broader socio-cultural context, such as how women started the use of ST, their attitudes/beliefs, and their knowledge of ST. Lastly, the well-informed framework, guided by the preceding literature review, along with the inductive and deductive approach of data analysis, was a great opportunity to compare what was already known and contrast at the same time with newer data from this study.

**Limitations.** Firstly, we were unable to interview the spouses and other family members of the pregnant women, which could have provided additional insights into the influence of family on ST use during pregnancy. Secondly, recruiting participants for the study was challenging due to the hesitancy of potential interviewees, possibly because of the involvement of CHWs who routinely engaged in tobacco control education. This recruitment process might have also introduced selection bias. Potentially, if the recruitment was independent of CHWs, the interviewees may have engaged in more effective conversations with the researcher. Thirdly, the environmental considerations with respect to the secret behaviour (especially parents) of women related to ST use, made it difficult to fully understand the family’s potential influence on their behaviour. However, this hidden aspect indirectly signifies family influence, challenging previous literature suggesting normality and acceptance of ST use among women. Lastly, due to resource limitations and feasibility constraints, our study only included eight in-depth interviews, and further research is warranted. Nevertheless, we reached data saturation, and no new themes emerged during the last few interviews.

### Implications for policy, practice, and further research

The overall perceptions and norms related to ST are starting to change, which of course is seen more now than before, it is likely to prevent women from initiating the experimental or peer-influenced use of ST, eventually preventing the cultivation of habit. Our study revealed that women tend to keep their use of ST a secret from family members while feeling more comfortable using it among peers who share a similar environment. Hence, increasing general awareness related to ST harms may be stigmatising its use and hence helping to reduce its prevalence among women. However, it is important that health promotion campaigns encourage the prevention and cessation of ST without stigmatising women using these products. Insights from tuberculosis interventions, which have successfully addressed stigma, can be applied to tobacco control efforts [31, 32]. Particularly, the framework outlined by Nutall and colleagues, which addresses stigma across different levels, including the general public, healthcare professionals, and individuals affected by tuberculosis, can serve as a valuable model for developing comprehensive tobacco control interventions aimed at tackling stigma from various aspects [31]. It is vital to address this barrier, as stigma isolates individuals and further hinders the ability to seek support for cessation services and lower the likelihood of successful quitting [33, 34]. Furthermore, the inclusion of CHWs to deliver tobacco control interventions should be considered. This could potentially be combined with community-based interventions or with the inclusion of family support. Furthermore, the study could have benefitted or further strengthened if women’s partners were also interviewed. This would have contributed to the perceptions and beliefs within the family and especially of the partners who may be key facilitators/barriers. Hence, this perhaps could be researched further to better inform tobacco control measures during pregnancy.

## Conclusion

No previous study has explored the use of ST during pregnancy from the perspective of pregnant women themselves. Our study sheds light on the influence of family and community/peers on ST use and reveal a shift in the norms and acceptance of this behaviour. These findings highlight the need for tobacco control measures to curb ST use among women in India and suggest a potential role for CHWs in supporting the prevention and cessation efforts. Furthermore, the recent shift in family attitudes may facilitate any efforts by health care workers to prevent ST use.

## Supporting information

S1 Checklist Inclusivity in global research. (DOCX)

S1 Table Topic guide. (DOCX)
