# Supplementary material for: How do family members influence smokeless tobacco consumption during pregnancy in India? Perspectives of pregnant women
Source: PLOS Glob Public Health. 2024 Jun 20;4(6):e0002828. doi: 10.1371/journal.pgph.0002828 (PMC11189192; doi:10.1371/journal.pgph.0002828)
Supplement: S1 Table — (DOCX) [file pgph.0002828.s002.docx]

S1 Table: Topic Guide

| **Topic** | **Questions** |
| --- | --- |
| Current ST use | What is your current ST use like? Can you please tell me the frequency and type of products you use? |
| ST and Pregnancy | Before pregnancy, what were your ST habits like? Has pregnancy changed any ST use of yours? |
|  | In what sense? Can you possibly tell me more about the change? |
|  | During the course of pregnancy, have there been any changes in your ST use? |
| ST initiation | When did you first use ST? |
|  | How did the habit start? |
|  | What factors influenced your use of ST? |
| Familial influence | What are ST habits in the family like? |
|  | How do those influence your ST behaviour? |
|  | How has the influence changed throughout the course of pregnancy? |
|  | How does you perceive these influences as? |
|  | Who amongst the family influences your behaviour the most? |
| Knowledge and accessibility | What according to you is ST? |
|  | How accessible are these products for you? |
|  | *Note: To be extra careful of not mentioning the words harm or danger to avoid the feeling of guilt and anxiety.* |

ST = smokeless tobacco
